# Salidroside ameliorates memory impairment following long-term ethanol intake in rats by modulating the altered intestinal microbiota content and hippocampal gene expression

**DOI:** 10.3389/fmicb.2023.1172936

**Published:** 2023-06-09

**Authors:** Yu Jiao, Zhenglin Zhao, Xin Li, Lulu Li, Dan Xiao, Siyuan Wan, Tong Wu, Tong Li, Ping Li, Rongjie Zhao

**Affiliations:** ^1^Department of Psychiatry, Qiqihar Medical University, Qiqihar, Heilongjiang, China; ^2^Department of Biochemistry, Qiqihar Medical University, Qiqihar, Heilongjiang, China; ^3^Department of Psychiatry, The Fourth Affiliated Hospital of Qiqihar Medical University, Qiqihar, Heilongjiang, China; ^4^School of Medicine and Health, Harbin Institute of Technology, Harbin, Heilongjiang, China; ^5^Department of Medicine and Health, Zhengzhou Research Institute of Harbin Institute of Technology, Zhengzhou, Henan, China; ^6^Department of Preventive Medicine, Qiqihar Medical University, Qiqihar, Heilongjiang, China

**Keywords:** Salidroside, ethanol, memory impairment, gut microbiota, hippocampus, metagenomics

## Abstract

**Background:**

Salidroside (*Sal*), the main component of a famous herb *Rhodiola rosea L*, enhances memory performance and reduces fatigue. Therefore, this study assessed the effect of *Sal* on memory impairment induced by a long-term intake of ethanol (EtOH) in rats and investigated its relevant mechanisms using gut microbiota metagenomic analysis and hippocampal transcriptomic analysis.

**Methods:**

Eighteen male SD rats were divided into the normal control group (CON group), EtOH model group (Model group), and *Sal* treatment group (*Sal* group). The rats in the Model and *Sal* groups intragastrically (i.g.) received 2 g/kg EtOH for 30 consecutive days, whereas the CON group was given an equal volume of distilled water. Meanwhile, the rats in the *Sal* group were administered i.g. 30 mg/kg *Sal* 60 min after EtOH intake. All rats were tested in the eight-arm maze for their memory function every 3 days. On the 30th day, metagenomic analyses of gut microbiota and transcriptomic analyses of the hippocampus were performed.

**Results:**

Compared with the Model group, *Sal* treatment reduced the total time to complete the eight-arm maze task, decreased the number of arm entries, and abated the working memory error that was significant from the 9th day. Additionally, *Sal* intervention improved the gut microbiota composition, such as the increased abundance of *Actinobacteria* and *Bifidobacterium*, which was related to the metabolism of amino acids and terpenoid carbohydrate, endocrine function, and signal transduction by neurotransmitters. In the hippocampus, the EtOH intake differentially expressed 68 genes (54 genes increased, whereas 14 genes decreased), compared with the CON group, whereas *Sal* intervention affected these changes: 15 genes increased whereas 11 genes decreased. And, enrichment analyses revealed these genes were related to the structural components of the ribosome, mRNA splicing process, protein translation, mitochondria function, and immunological reaction. Finally, a correlation analysis found the memory impairment was positively correlated with the abnormal upregulation of *Tomm7* but negatively correlated with decreased abundance of gut *Alistipes_indistinctus*, *Lactobacillus_taiwanensis*, *Lactobacillus_paragasseri*, and *Lactobacillus johnsonii*.

**Conclusion:**

*Sal* improved memory impairment caused by long-term EtOH intake in rats, which may be related to its regulation of gut dysbiosis and hippocampal dysfunction.

## Introduction

In recent times, long-term alcohol use has imposed a tremendous disease burden on human health worldwide. Long-term alcohol use is associated with more than 200 diseases (Dumont et al., 2020; Ke et al., 2022). The long-term alcohol syndrome has a broad spectrum of symptoms, including severe memory dysfunction that seriously affects the quality of life of alcohol abusers. Being a small organic molecule, ethanol (EtOH) readily passes through different biomembranes, including the blood–brain barrier. More than 99% of the about 100 billion resident neurons in the brain are either glutamatergic (excitatory) or GABAergic (inhibitory) neurons, and they are linked together to construct complex networks and structures to implement appropriate functions of the brain, including learning and memory. EtOH is both an allosteric agonist of GABAa receptors and allosteric antagonist of the ionotropic glutamatergic receptors, indicating that most alcohol abusers suffer from certain kinds of memory impairment (Parsons, 1986). However, the mechanisms by which alcohol consumption causes impaired memory function have not been fully elucidated.

Long-term alcohol use leads to neurological dysfunction in different brain regions, such as prefrontal cortex, hippocampus, and amygdala, which are involved in memory processes (Topiwala et al., 2017; Caleb et al., 2022; Collin and Eric, 2022). The hippocampus is the most significant structure among the aforementioned brain regions, because it is the primary structure for encoding information and memory formation. Additionally, it is involved in almost every aspect and process of memory, such as the consolidation of information from short-term memory to long-term memory, formation of spatial and working memory, and memory extinction and reconsolidation. Moreover, a growing number of studies have shown that the hippocampus is susceptible to damage by long-term EtOH use. Memory impairment in alcohol abusers seems to greatly depend on the hippocampus (Contreras et al., 2019).

Long-term alcohol intake leads to disturbed gastrointestinal function, which can cause diverse symptoms in the body, including high cognitive dysfunction (Choi, 2021; Zhao et al., 2021). The gut microbiota and the central nervous system (CNS) have bidirectional connections with respect to the anatomy, physiology, and biochemical interactions. The gut–brain axis is involved in almost every facet of the physiologic processes of the body, such as neural, endocrine, and immune responses (Xia et al., 2021). For instance, the microbiota induce and produce neurotransmitters, hormones, and other physiological factors either by themselves or by stimulating gastrointestinal cells, which together with their neuroactive metabolites enter the circulatory system, regulating CNS functions, including learning and memory (Marcondes Ávila et al., 2020; Pradhananga et al., 2020). In turn, the CNS controls the gut microbiota through various biological signaling molecules, such as hormones, cytokines, and neurotransmitters, affecting the composition and diversity of gut microbiota, which complete the cyclic regulation of the CNS-gut-CNS circle (Cox and Weiner, 2018). Therefore, the effect of long-term consumption of EtOH on gut microbiota and its critical role in regulating learning and memory have been the focus of research (Qamar et al., 2019).

Currently, the main drugs that are widely prescribed in clinical practice for treating cognitive impairment—including memory dysfunction—are pyranacetam (Uniyal et al., 2019), anisacetam, and other brain function improvement agents. These drugs not only improve cognitive dysfunction but also possess therapeutic effects on cerebrovascular disease, brain trauma, vascular dementia, and Alzheimer’s disease. However, their effects on alcoholic memory impairment are limited. They have severe side effects, such as dry mouth, nausea, vomiting, abdominal discomfort, and insomnia, which greatly compromise their clinical use. Therefore, the development of safe herbal or alternative drugs with fewer side effects has become increasingly urgent. *Rhodiola rosea L* is a perennial herb, which has therapeutic effects on mental disorders, such as anxiety and depression (van Diermen et al., 2009). Salidroside (*Sal*) is the main component of *Rhodiola rosea L*, and like its parent herb, it can enhance mental strength and reduce fatigue (Shevtsov et al., 2003). For example, *Sal* increases the CNS levels of some neurotransmitters, such as norepinephrine (NE), dopamine (DA), serotonin (5-HT), and acetylcholine (Ach), to stimulate CNS activity, especially enhance learning and memory (Panossian et al., 2008; Li et al., 2017). However, no study on the effect of *Sal* on memory impairment induced by long-term alcohol use has yet been reported.

Taken together, the above evidence points to a possible therapeutic role of *Sal* on memory impairment caused by a long-term EtOH consumption. Therefore, in the present study, we established a long-term EtOH intake rat model and evaluated *Sal* effects on memory impairment using the eight-arm maze (EAM) test. After the confirmation of *Sal* therapeutic effects, the relevant mechanisms were explored by metagenomic analyses of gut microbiota and the hippocampus. Additionally, a triple correlation analysis was performed between behavior and gut microbiota changes and hippocampal gene expression changes. This work not only provides a basic research evidence for the development of Sal as a promising candidate and potential strategy to treat memory deficit in alcoholic conditions, but also address the correlation between the gut microbiota and the hippocampus functionally interact to mediate memory impairment caused by a long-term ethanol consumption.

## Materials and methods

### Establishment of alcohol-induced memory impairment model in rats and intervention with *Sal*

Eighteen SD rats (male, 180–220 g) were purchased from the Animal Laboratory Center of Qiqihar Medical University. All rats were individually housed with controlled diet and free access to water on a 12-h dark/light cycle at a constant humidity of 55 ± 5% and temperature of 22 ± 2°C. All experimental protocols were approved by the Animal Ethics Committee of Qiqihar Medical University (QMU-AECC-2023-87). The alcoholism modeling method was slightly modified based on the experiment of Jiao Yu et al. (2021). All experimental protocols were approved by the Animal Ethics Committee of Qiqihar Medical University. The alcoholism modeling method was slightly modified based on the experiment of Sase et al. (2016). Rats (six rats in each group) were randomly divided into the control group (CON group), chronic alcoholism model group (Model group), and *Sal* group. Briefly, in the subsequent 30 days, the Model and *Sal* groups were continuously administered with EtOH (2 g/kg/day, dissolved in distilled water) by the intragastric (i.g.) route. The *Sal* group was administered with *Sal* (30 mg/kg/day, i.g., dissolved in distilled water) 60 min after alcohol intake, whereas the CON and Model groups were given the same volume of distilled water. Memory was assessed by the EAM behavior experiment on days 3, 6, 9, 12, 15, 18, 21, 24, 27, and 30 of the experiment. *Sal* (purity ≥98%) was purchased from Shaanxi Wanyuan Biotechnology Co (Xi an, Shan Xi, China).

### Cognitive status measurement

Cognitive Status Measurement was slightly modified based on the experiment of Sase et al. (2016). The EAM test consisted of eight radiation arms and a central platform. Eight arms radiate from an octagonal platform in the middle, each 50 cm long. The angle between the arms was 45°. A 30-cm-high baffle was placed at the beginning of each arm, and eight baffles blocked the central platform. A thermal imager was placed 180 cm above the platform and was responsible for recording data, such as the movement trajectory of the rat and number of entries into each radiation arm throughout the experiment. The entire maze was made of black organic plastic. Throughout the experiment, the maze was placed in the center of the lab at a fixed position, and the position of other objects in the lab remained unchanged.

Rats were trained once a day for 7 days prior to testing, while maintaining 80% of their normal feeding throughout the experiment. At the beginning of training, rats were placed on the central platform of the maze for 15 s, followed by removal of the eight radiating arm baffles. Rats freely chose to enter the end of the radiated arm for foraging for 10 min or completed foraging for all arms prematurely, indicating the end of training. Following each training session, the maze was wiped with acetic acid to eliminate the influence of odors on subsequent testing. Food was placed only at the end of arms 1, 2, 5, and 7 (working arm), and no food was placed in arms 3, 4, 6, and 8 (reference arm) throughout the test. The order of placement of the entire food arm remained unchanged in this experiment. Repeated entries into the working arm were recorded as working memory errors (WMEs).

Test indicators were as follows:

(1) Total time for rats to complete all working arms of the eight-arm maze: time to pass arms 1, 2, 5, and 7. If not completed within 10 min, time was recorded as 10 min. (2) Total number of working memory errors: the total number of errors within 10 min; error was defined as repeated entry into the same working arm. (3) Total number of arm entries: total number of arm entries of rats in a test.

**Figure 1:**
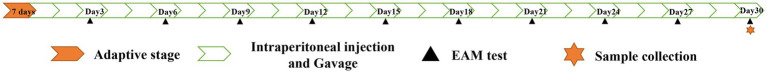
Experimental time flow diagram. EAM test, eight-arm maze detection.

Changes were detected and analyzed on experimental days 3, 6, 9, 12, 15, 18, 21, 24, 27, and 30. The specific experimental flow is shown in Figure 1.

### Hippocampal and intestinal content collection

At the end of the behavioral testing on day 30, the rats were decapitated immediately to collect blood samples. Rat skull was dissected, and the hippocampus was rapidly collected. Additionally, 2 g of intestinal contents were collected from all rats and rapidly frozen in liquid nitrogen and transferred to a −80°C freezer. All operations were performed on a sterile ice bath.

### Metagenomic detection of intestinal contents

The metagenomic sequencing of intestinal contents was performed according to this order: sample DNA extraction, library sequencing, data quality control, species annotation and analysis, gene set construction and analysis, functional annotation analysis, and other processes. In this project, metagenomic analysis was performed using the Wekemo Bioincloud (Shenzhen, China).^1^ Specific steps were as follows:

① Sample extraction testing and sequencing of intestinal flora

Genomic DNA was extracted from intestinal content samples using the CTAB method. Libraries were constructed using the NEB Next ^®^ Ultra ^™^ DNA Library Prep Kit for Illumina (NEB, Ipswich, United States). Qualified DNA samples were randomly interrupted into fragments of approximately 350 bp in length using a Covaris ultrasonic disruptor (Covaris S2 System, Massachusetts, United States), and the entire library preparation was completed by end repair, deployment A tail, sequencing adaptor, purification, and PCR amplification of the DNA fragments. Finally, the AMPure XP system purified the PCR products, detected insert size of the library using Agilent2100 (Agilent Technologies Co., Ltd., United States), and quantified library concentration using real-time PCR (Bio-Rad, United States). Indexed coding samples were clustered on the cBot Cluster Generation System using the Illumina PE Cluster Kit (Illumina, United States). Following cluster generation, DNA libraries were sequenced on the Illumina Novaseq 6000 (Illumina, San Diego, CA, United States) platform, and 150 bp double-ended reads were generated.

② Data analysis

Data quality control and de-host sequence: Metagenomic sequencing was performed using the Illumina NovaSeq high-throughput sequencing platform to obtain metagenomic raw data for bacteria, fungi, and archaea in intestinal content samples. Raw sequencing data were pre-processed using the Knead data software.

③ Species and functional annotations

Kraken2 and self-built microbial nucleic acid databases (screened NCBI NT nucleic acid database and RefSeq whole genome database for sequences belonging to bacteria, fungi, ancient fine bacteria, and viruses) were aligned to calculate the number of sequences containing species in each group of samples, and then Bracken was used to predict the actual relative abundance of species in the samples. Sequences after quality control and de-hosting were aligned (DIAMOND based) to the Protein Data Bank (UniRef90) using the HUMAnN2 software. PCoA analysis was based on specie abundance tables and functional abundance tables. Lefse analysis and mining were performed to detect differences in species composition and functional composition between samples (Villar et al., 2015).

④ Resistance gene annotation

The DIAMOND software (Buchfink et al., 2015) was used to align quality control and de-hosted sequences of each sample to the CARD database, and the sequences that failed the alignment were filtered out [parameters: -e 0.001 (*e*-value <1e-3) -i80 (identity percent >80%)]. The relative abundance of antibiotic resistance genes in each group of samples was obtained from the alignment (Detailed results are shown in the Supplementary Figure S1).

### Hippocampal transcriptome sequencing analysis

Using the Illumina sequencing platform, all RNAs of the samples were sequenced, and the high-quality data were compared with the reference genome for further analysis of expression quantification, differential genes, and functional annotation. In this study, transcriptome biochemistry analysis was performed using the Wekemo Bioincloud (see text footnote 1) (Shenzhen, China).

① Sample collection and preparation

RNA was extracted using standard extraction methods, and RNA samples were tested for RNA integrity using the Agilent 2100 bioanalyzer (Agilent Technologies Co. Ltd., United States) for quality control. The mRNA with polyA tails was enriched by Oligo(dT) magnetic beads using the NEBNext^®^ UltraTM RNA Library Prep Kit (Illumina), and the resulting mRNA was subsequently randomly interrupted with divalent cations in the NEB Fragmentation Buffer. The fragmented mRNA was used as a template and random oligonucleotides as primers to synthesize the first strand of cDNA in the M-MuLV reverse transcriptase system, followed by degradation of the RNA strand with RNaseH and synthesis of the second strand of cDNA with dNTPs under the DNA polymerase I system. The purified double-stranded cDNA was end-repaired, A-tailed, and connected to the sequencing junction. The cDNA of 250–300 bp was screened by AMPure XP beads and amplified by PCR, and the PCR products were purified by AMPure XP beads again to obtain the library. After library construction, initial quantification was performed using a Qubit 2.0 Fluorometer (Thermo Scientific, United States), and the insert size of the library was subsequently checked using an Agilent 2100 bioanalyzer. After the libraries passed the test, Illumina sequencing was performed to generate 150 bp paired-end reads.

② Data quality control and differential expression analysis

To ensure the quality and reliability of data analysis, filtration of raw data is necessary. It mainly includes removal of reads with adapter, removal of reads containing N (N indicates that base information cannot be determined), and removal of low-quality reads (reads with Qphred ≤20 bases accounting for more than 50% of the entire read length). Additionally, clean data were subjected to Q20, Q30, and GC content calculation. All subsequent analyses were based on clean data for high quality analysis. Indexing of the reference genome was constructed using HISAT2v2.0.5, and paired-end clean reads were aligned to the reference genome using HISAT2 v2.0.5. StringTie (1.3.3b) for novel gene prediction. Differential expression analysis between the two compared combinations was performed using the DESeq2 software (1.16.1).

③ Enrichment and pathway analysis

GO enrichment analysis of differentially expressed genes (Gene Ontology, GO) was performed using the clusterProfiler (3.4.4) software. Statistical enrichment of differentially expressed genes in KEGG pathways was analyzed using the clusterProfiler (3.4.4) software.

### Data processing and statistical analysis

All experiments were repeated three times. Data are presented as the mean ± standard error of the mean (SEM). One- or two-way ANOVA followed by Tukey’s post-hoc test was used for multiple comparisons. Data were analyzed using GraphPad Prism 8.0 software (GraphPad Software, Inc.). Correlation analyses were performed using R software (V4.2.2).

## Results

### Improving the effect of *Sal* on memory dysfunction in chronic EtOH intake rats

**Figure 2:**
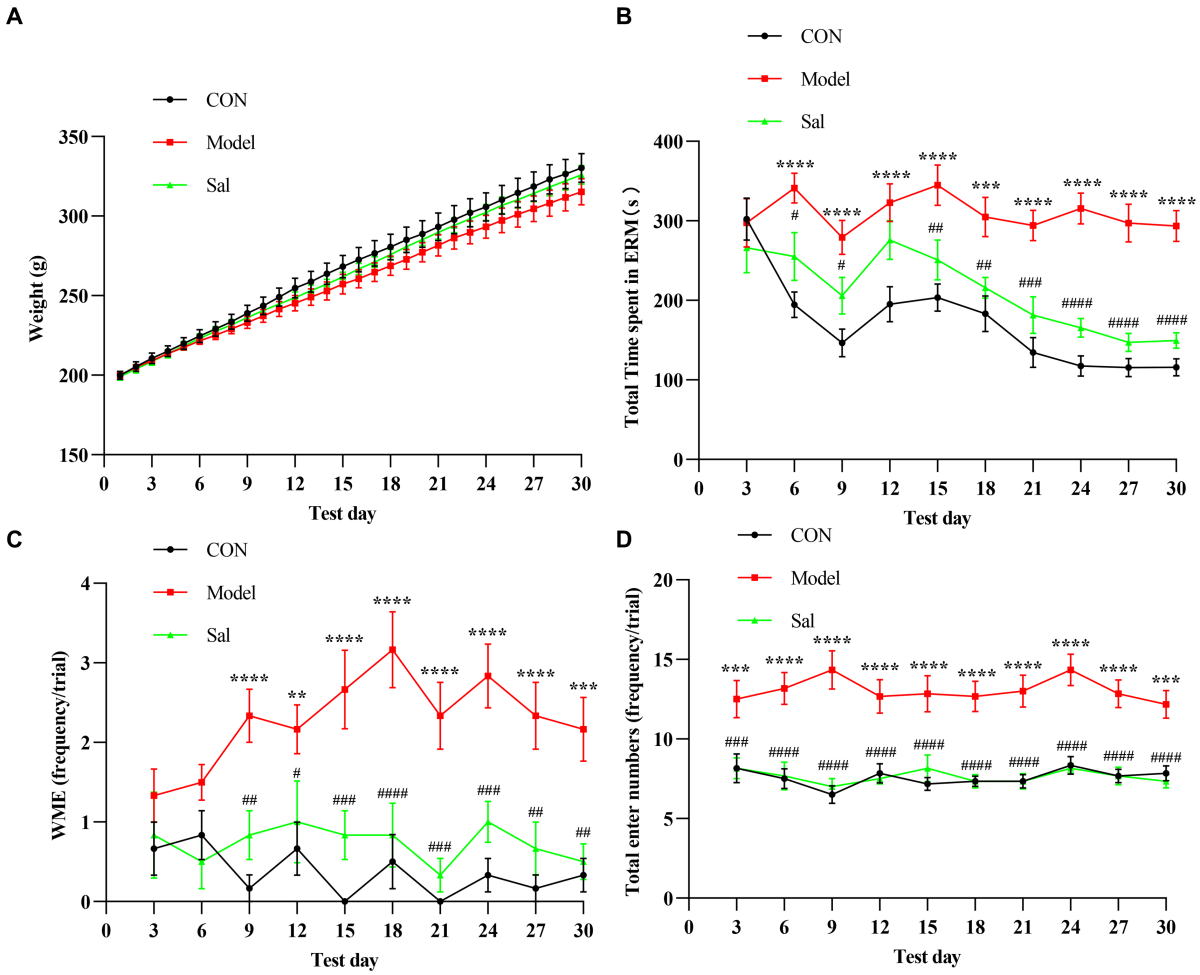
*Sal* improves the memory function during alcoholism in rats. **(A)** There was no significant difference in body weight among CON, Model, and *Sal* groups. **(B–D)** The total maze time, the number of working memory errors, and the total number of arm entries were significantly increased in the Model group compared with the other two groups. Two-way ANOVA and Tukey analyses were used for multiple group comparison. Data are shown as mean ± SEM, *n* = 6 in each group. ^**^p < 0.01, ^***^p < 0.001, ^****^p < 0.0001 (CON vs. Model); ^#^*p* < 0.05, ^##^*p* < 0.01, ^###^*p* < 0.001, ^####^*p* < 0.0001 (Model vs. *Sal*).

In successive experiments over 30 days, no apparent difference in body weight was observed among the groups (Figure 2A). Cognitive function was measured using the EAM test, and the total time to complete the EAM was generally higher in the Model group than in the other two groups. Rats in the *Sal* group were more effective during the EAM task (*p* < 0.001, Figure 2B). Compared with the CON group, the Model group had WMEs that began to rise on day 9 (*p* < 0.01), whereas the *Sal* group had a decreased number of WMEs (*p* < 0.01, Figure 2C). Additionally, the total number of arm entries in the Model group was significantly more than that of the CON group (*p* < 0.001), whereas *Sal* intervention reduced the number of arm entries (*p* < 0.001, Figure 2D). Taking these results together, we concluded that chronic administration of alcohol caused working memory impairment, whereas administration of *Sal* improved memory function against alcoholic consumption.

### *Sal* treatment affects intestinal diversity of microbes by metagenomic analysis of gut contents

#### Sequence control and de-host sequences

Double-end sequencing was performed using the Illumina sequencing platform. Raw data were obtained by sequencing. The pre-processed protocol was as follows: ① the linker sequence was removed (parameter ILLUMINACLIP: adapters_path: 2:30:10); ② the scanned sequence (4-bp sliding window size). The removal condition of subsequent sequence was set as average mass score < 20 (99% accuracy, parameter SLIDINGWINDOW: 4:20); and ③ the sequence with a final length of <50 bp was removed (parameter MINLEN: 50) (see Supplementary Table S1 for detailed results).

### Analysis of sample composition

**Figure 3:**
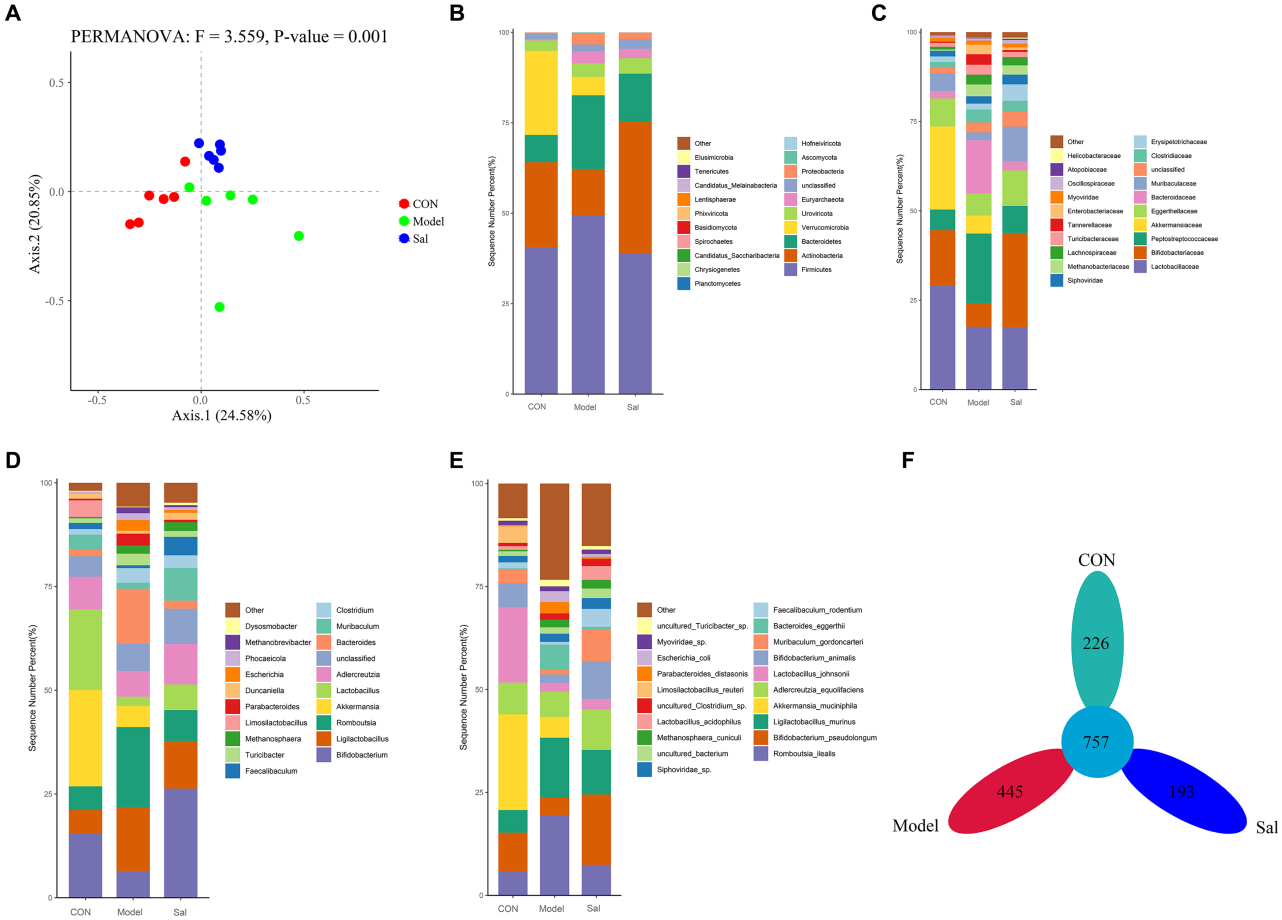
Composition of the gut microbiota in the CON, Model and Sal groups. **(A)** Bray-Curtis-based PCoA plots that can relatively effectively separate the Model group from the remaining two groups, indicating that the gut flora composition of the Model group is significantly different from the remaining two groups. Distribution characteristics of the gut microbiota in the CON, Model and Sal groups (*n* = 6 for each group). Histograms of relative abundance of the top 20 species at the **(B)** phylum level, **(C)** family level, **(D)** genus level, and **(E)** species level among the three groups with Wilcoxon rank-sum test. **(F)** Petal plots of the three groups.

The diversity among samples was analyzed by principal component analysis (PCoA). If the samples were similar in species composition, the closer they were in the PCoA plot. The results showed (Figure 3A) that Axis 0.1 indicated 24.57% differentiation and Axis 0.2 indicated 20.88% differentiation (*F* = 3.557, *p* = 0.001). Compared to the relatively scattered distribution in the Model group, the distributions in the CON and *Sal* groups were more concentrated, indicating that *Sal* intervention had an effect on the composition of the intestinal flora of rats in the model of alcohol-induced memory damage and brought the composition of the intestinal flora close to that of the control group. All valid sequences of the samples were annotated and classified by Kraken2 (Wood and Salzberg, 2014) (parameter –confidence 0.2), and the proportion of the number of sequences in the samples at the Kingdom level to the total number of sequences were performed. The species detected were Archaea (1.56%, 1,276,538), Bacteria (95.21%, 77,692,265), Fungi (0.13%, 105,304), Heunggongvirae (2.86%, 2,333,896), and Viruses (0.23%, 187,864).

Bacteria accounted for the largest number of sequences. At the phylum level, the *Sal* group was richer in the Actinobacteria phylum than the other two groups, and the Model group had a higher relative abundance of the thick-walled phylum and a lower relative abundance of the Actinobacteria phylum. At the family level, the Lactobacillus family was more abundant in the CON group than the other two groups. At the genus level, the genus Bifidobacterium was significantly more abundant in the *Sal* group than in the Model group. At the species level, *Romboutsia ilealis* was more abundant in the Model group than the CON and Sal groups. A total of 757 OTUs were detected in the three groups; 226, 445, and 193 OTUs were specific to the control, Model, and *Sal* groups, respectively (Figures 3B–F). The taxonomic levels of archaebacteria and fungi are shown in Supplementary Figure S2.

### Differential microbial screening

**Figure 4:**
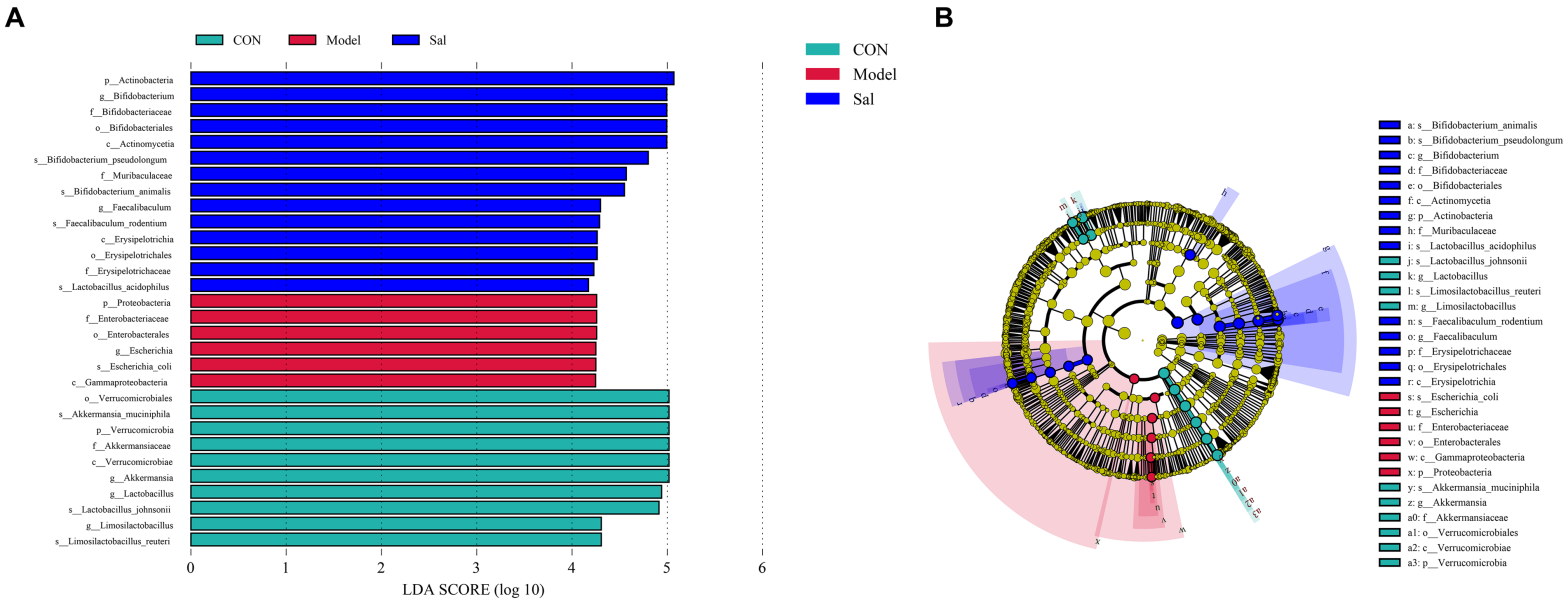
Linear discriminant analysis (LEfSe) among the CON, Model, and Sal groups, LDA = 4.

The characteristic microorganisms of each group were determined using the LEfSe analysis (linear discriminant analysis effect size method), and the relationships between different microbial groups, from phylum to species level (LDA = 4), are shown in the branching diagram (Figures 4A,B). The results showed that the 30 OTUs at the phylum (3 OTUs), order (4 OTUs), order (4 OTUs), family (5 OTUs), genus (6 OTUs), and species levels (8 OTUs) differed significantly among the three groups. The relative abundance of Proteobacteria, Enterobacteriaceae, Enterobacterales, Escherichia, Escherichia_coli, and Gammaproteobacteria was higher in the Model group. In the control group, Verrucomicrobiales, Akkermansia_muciniphila, Verrucomicrobia, Akkermansiaceae, Verrucomicrobiae, Akkermansia, Lactobacillus Lactobacillus_johnsonii, Limosilactobacillus, and Limosilactobacillus_reuteri were more abundant. In the *Sal* group, Actinobacteria, Bifidobacterium Bifidobacteriaceae, Bifidobacteriales, Actinomycetia, Bifidobacterium_pseudolongum, Muribaculaceae, Bifidobacterium_animalis Faecalibaculum, Faecalibaculum_rodentium, Erysipelotrichia, Erysipelotrichales, Erysipelotrichaceae, and Lactobacillus_acidophilus had higher relative abundance.

### Functional analysis prediction

**Figure 5:**
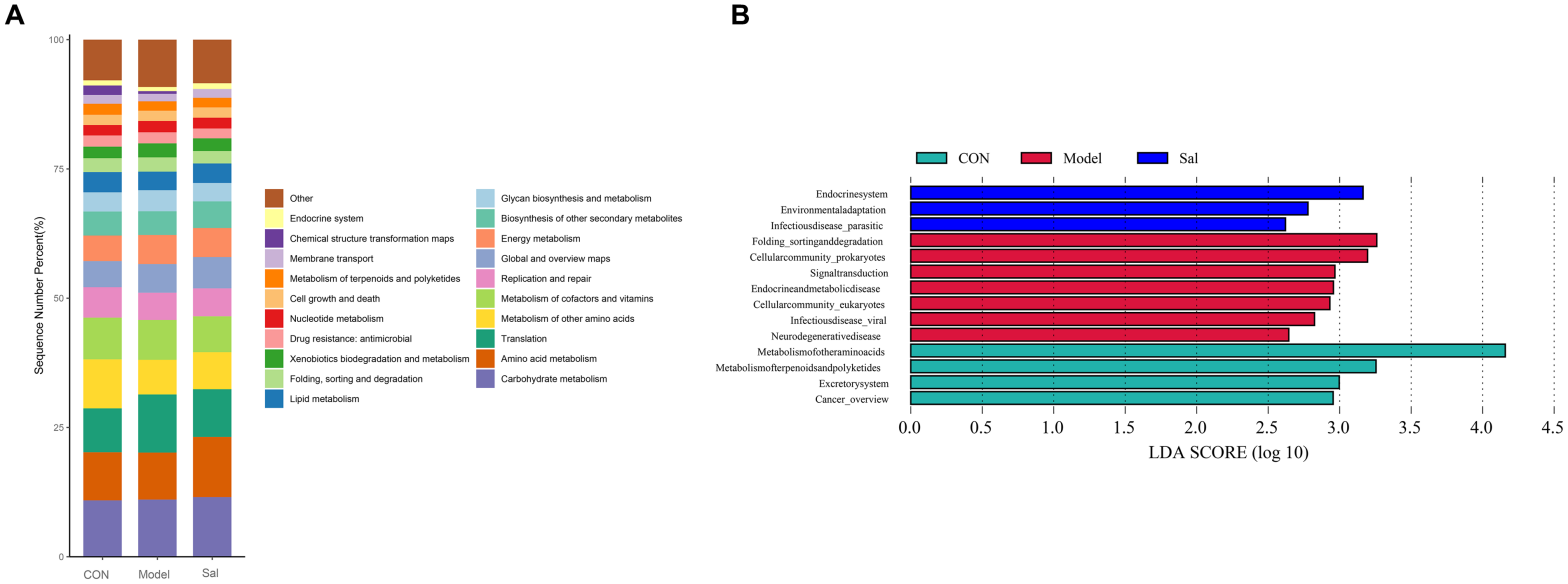
KEGG pathway analysis among the CON, Model, and Sal groups. **(A)** Secondary metabolic pathway map. **(B)** Results of LEfSe analysis (LDA = 2).

Based on the search of the KEGG (Kyoto Encyclopedia of Genes and Genomes) database for comparative annotations, a KEGG secondary pathway map was obtained (Figure 5A). No significant difference in the secondary pathway was observed among the three groups of samples. The LDA bar graph was obtained by LEfSe analysis of the basic metabolic pathways of KEGG (set LDA = 2), and the results are shown in Figure 5B. In the CON group, the metabolic pathways involved were the metabolism of other amino acids and metabolism of terpenoids and polyketides. Additionally, Excretorysystem and Cancer_overview were relatively high. The Model group mainly involved seven highly expressed metabolic pathways, including Folding_sorting and degradation, Cellular community_prokaryotes, Signal transduction Endocrine and metabolic disease, Cellular community_eukaryotes, Infectious disease_viral, and Neuro degenerative disease. In the *Sal* group, three highly expressed metabolic pathways were involved: Endocrine system, Environmental adaptation, and Infectious disease_parasitic.

### *Sal* treatment affects hippocampal transcriptomics in alcohol consumption rats and data quality control and variance expression analysis

Quality control processing was performed on the raw data of each sample using the fastp software (Chen et al., 2018) to obtain clean data. A total of 273,318,968, 274,582,288, and 284,832,284 clean reads were generated from the control, Model, and Sal groups, respectively. Where the Q20 values were all higher than 97%, the Q30 values were all higher than 92%, the error rate was 0.03, and the GC content distribution was about 48% (Supplementary Table S2 for detailed results). The clean data reads after QC were compared to the reference genome using HISAT2 (Kim et al., 2015), and the comparison was evaluated using Qualimap RNA-seq (Konstantin et al., 2016). A total of 210,235,503, 211,159,294, and 220,176,485 reads were generated from the control, Model, and *Sal* groups mapped to the rat genome, respectively. Differentially expressed genes were screened by screening out detection rates (proportion of count not 0) <0.25, |log 2 (Fold Change)| > 1 & padj<0.05. The results revealed 68 differentially expressed genes in the Model group, compared with the CON group. Among them, 54 genes had elevated relative expression and 14 genes had decreased relative expression.

**Figure 6:**
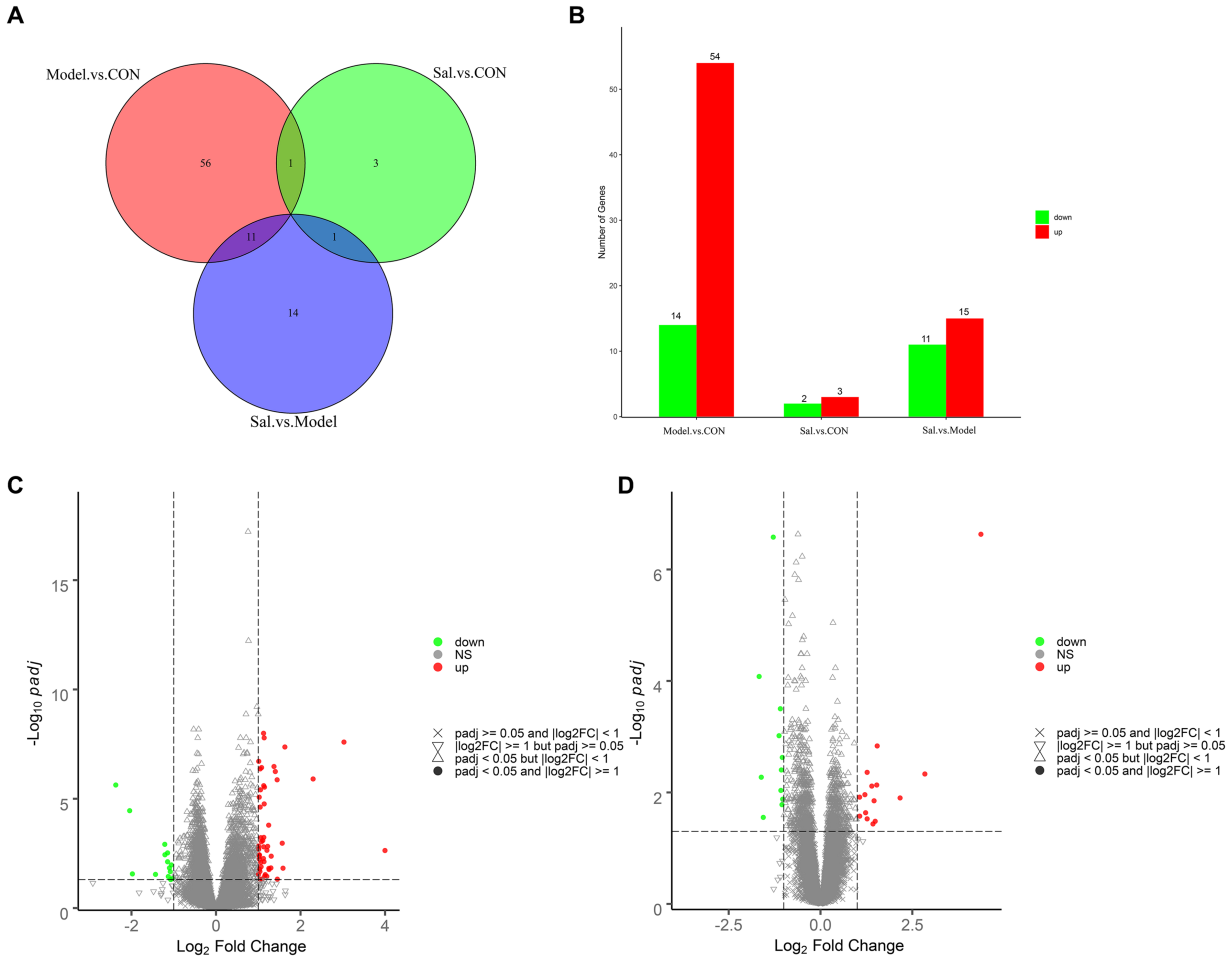
Analysis of differentially expressed genes among the CON, Model, and Sal groups. **(A)** Venn diagram of groups. **(B)** Histogram of differentially expressed genes among groups. **(C)** Volcano diagram of differentially expressed genes between the Model and CON groups. **(D)** Volcano diagram of differentially expressed genes between the Sal and Model groups. Red and green dots indicate up- and down-regulation, and gray dots indicate no differential gene expression.

The top five relatively highly expressed genes were Cnmd (log2Fold Change = 4.00, padj = 2.33E-03), S100a9 (log2Fold Change = 3.03, padj = 2.55E-08), Tomm7 (log2Fold Change = 1.63, padj = 4.40E-11), Klhl40 (log2Fold Change = 1.57, padj = 1.08E-03), and Lilrb3a (log2Fold Change = 1.45, padj = 4.77E-02). The first five relatively low-expressing genes were AY172581.16 (log2 Fold Change = −2.37, padj = 2.35E-06), AY172581.17 (log2Fold Change = −2.04, padj = 3.51E-05), Npsr1 (log2Fold Change = −1.98, padj = 2.74E-02), AY172581.5 (log2Fold Change = −1.43, padj = 2.88E-02), and Rxfp1 (log2Fold Change = −1.21, padj = 1.22E-03). Twenty-six differentially expressed genes were identified in the Sal group, compared to the Model group. Among these genes, 15 genes were elevated and 11 genes were decreased. The top five relatively highly expressed genes were Olr1694 (log2Fold Change = 2.84, padj = 4.67E-03), Ca3 (log2Fold Change = 2.17, padj = 1.26E-02), Synpo2 (log2Fold Change = 1.54, padj = 1.47E-03), AY172581.17 (log2Fold Change = 1.53, padj = 7.37E-03), and Spp1 (log2Fold Change = 1.49, padj = 3.29E-02). The top five relatively lowly expressed genes were Ch25h (log2Fold Change = −1.56, padj = 2.80E-02), Tubb2b (log2Fold Change = −1.29, padj = 2.63E-07), Tomm7 (log2Fold Change = − 1.13, padj = 9.61E-04), Naa38 (log2Fold Change = −1.09, padj = 3.15E-04), and Mei1 (log2Fold Change = −1.08, padj = 9.21E-03). The results are shown in Figures 6A–D.

### GO pathway analysis

**Figure 7:**
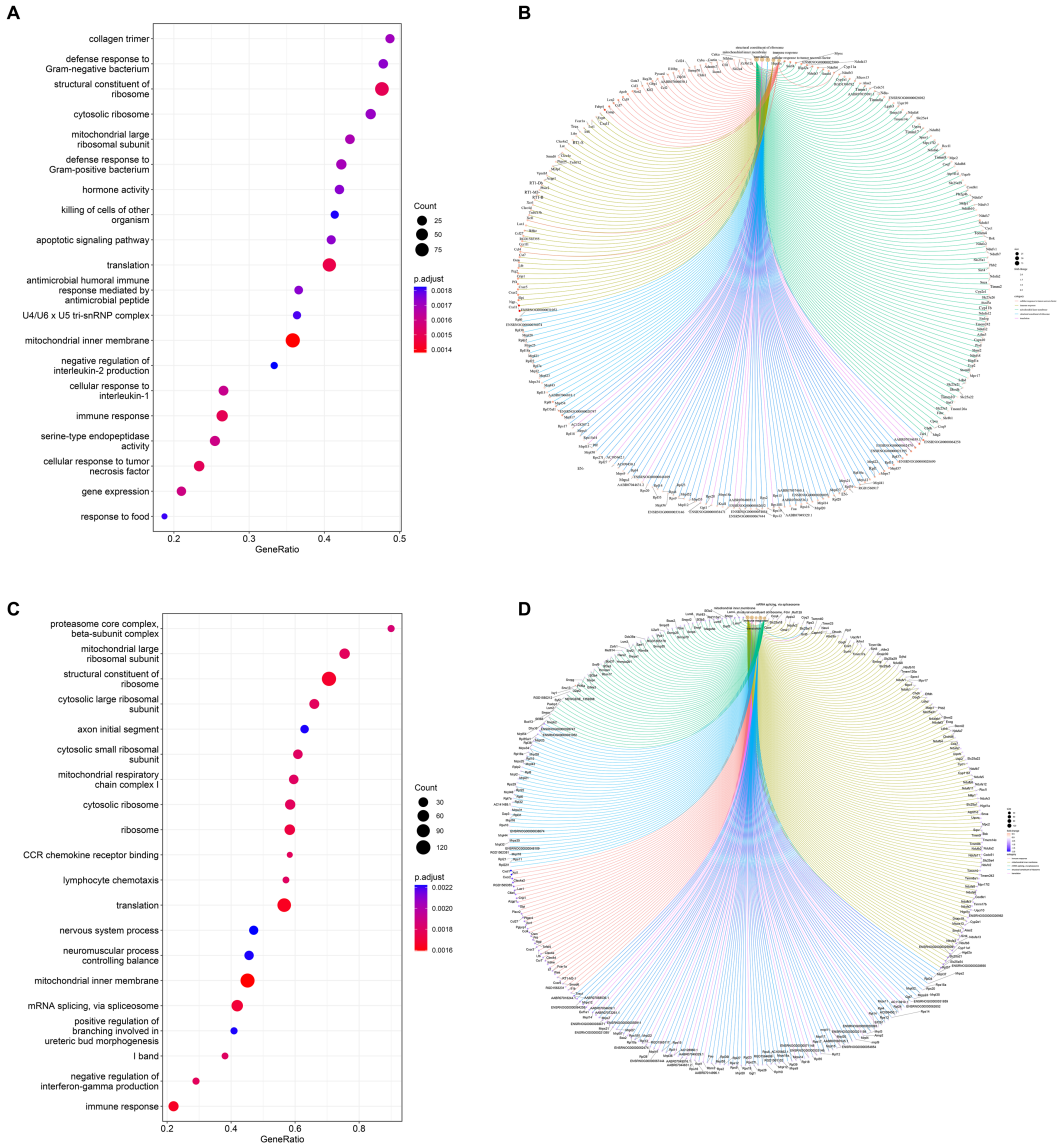
Dot plots of the main GO term enrichment analysis and core_enrichment gene plots (*n* = 6 per group). Comparison of the 20 most important terms between **(A)** the Model and CON groups and **(C)** the Sal and Model groups. **(B)** Plots of core genes enriched to key terms between the Model and CON groups and **(D)** Sal and Model groups, with each dot representing a core gene.

Based on the GO database, the gene set enrichment analysis (GSEA) of the function of genes among the three groups revealed 314 GO terms with significant differences between the Model and CON groups. They included biological processes (197 subclasses), cell components (57 subclasses), and molecular functions (60 subclasses). Among them, the top five terms included mitochondrial inner membrane, translation, structural constituent of ribosome, immune response and cellular response to tumor necrosis factor (Figure 7A). The core genes related to each term are shown in Figure 7B. A total of 652 significant GO terms were found between the *Sal* and Model groups; they included biological processes (416 subclasses), cell components (122 subclasses), and molecular functions (114 subclasses). Among them, the top five terms were mitochondrial inner member, translation, immediate response, structural constitution of ribosomes, and mRNA splicing, *via* spliceosome (Figure 7C). The core genes related to each term are shown in Figure 7D.

### Combined multi-omics correlation analysis

**Figure 8:**
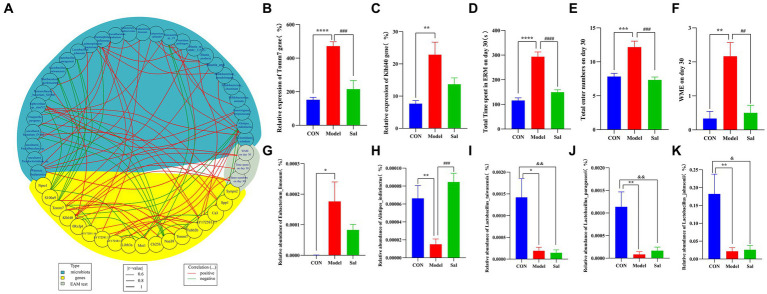
Spearman’s correlation analysis was performed for behavioral, enterobacterial genome and hippocampal transcriptome difference data. **(A)** Circle plot of behavioral, genomic and transcriptome difference data (blue represents differential microorganisms, yellow represents differential genes, gray represents behavioral; red line represents positive correlation, green line represents negative correlation). **(B)** Relative expression level of differential gene *Tomm7* in hippocampus [^****^p < 0.0001 (CON vs. Model); ^###^*p* < 0.001 (Model vs. Sal)]. **(C)** Relative expression of the differential gene *Klhl40* in the hippocampus [^**^p < 0.01 (CON vs. Model)]. **(D–F)** Total time of eight-arm maze test, total number of arm entries, and WME [^**^p < 0.01, ^***^p < 0.001, ^****^p < 0.0001 (CON vs. Model); ^##^*p* < 0.01, ^###^*p* < 0.001, ^####^*p* < 0.0001 (Model vs. Sal)]. **(G–K)** Relative abundance of differential microorganisms *Eubacteriumlimosum*, *Alistipesindistinctus, Lactobacillustaiwanensis*, *Lactobacillusasseri*, and *Lactobacillusjohnsonii* [^*^p < 0.05, ^**^p < 0.01 (CON vs. Model); ^###^*p* < 0.001 (Model vs. Sal); ^&^*p* < 0.05, ^&&^*p* < 0.01 (CON vs. Sal)].

Correlation analyses among data of behavioral, microbial genome, and hippocampal transcriptome were performed using the R package(R 4.2.2) (Figure 8). Compared with CON group, the Model group had a total time to complete the EAM, total number of arm entries, and WMEs on the 30th day to be positively correlated with the upregulation of *Tomm7* and *Klhl40* mRNA in rat hippocampus. The upregulation of *Tomm7* and *Klhl40* mRNA was positively correlated with the abundance of *Eubacteriumlimosum* and negatively correlated with the abundance of *Alistipesindistinctus*, *Lactobacustaiwanensis*, *Lactobacusparagi*, and *Lactobacusjohnsonii* in the intestine of the Model group. Compared with the Model group, the *Sal* group had a significantly decreased mRNA expression of *Tomm7* in the hippocampus upon administration of *Sal*. This decreased expression was positively correlated with the abundance of *Alistipesindistinctus*, *Lactobacustaiwanensis*, *Lactobacusparagi*, and *Lactobacusparagillusjohnsonii* in the intestine of the *Sal* group.

## Discussion

In this study, we observed the ameliorative effect of *Sal* on memory impairment caused by long-term administration of EtOH in rats, which was manifested as the reduction in total time to complete the EAM task, decreased number of arm entries, and abated working memory errors in the EAM tests. Additionally, the metagenomic analysis revealed that the composition and diversity of intestinal flora were significantly altered in rats with memory improvement after *Sal* intervention, which are mainly associated with metabolism of amino acids, terpenoids, and polyketides. For instance, EtOH caused a decrease in the relative abundance of *Actinobacteria, Bifidobacteriaceae, Akkermansiaceae*, and *Lactobacillus* and an increase in the relative abundance of *Romboutsia ilealis* in the intestinal tract of rats. The relative abundance of microorganisms in their intestinal contents improved with *Sal* intervention, especially the relative abundance of *Bifidobacterium, Ligilactobacillus, Adlercreutzia*, and *Lactobacillus* was increased. These findings confirmed the effect of *Sal* on intestinal microbes.

To verify whether *Sal* acts on the gut-brain axis, a transcriptomic analysis of hippocampal tissue was then performed. The overall results showed that 26 genes were differentially expressed after *Sal* intervention, compared with the Model group. Furthermore, enrichment analyses revealed that these differential genes were mainly involved in the mitochondrial inner membrane, translation, immune response, structural constituent of ribosome, and mRNA splicing. Finally, the multiple correlation analyses uncovered the correlation among the behaviors, gut microbiota, and changed expression of hippocampal genes. Altogether, these findings provide evidence that *Sal* can attenuate alcoholic memory impairment, which may be associated with an improvement in gut-brain axis function.

Memory is the physiological process of the CNS by which information or data about intra- or extra-environments are encoded, stored, and retrieved when needed. Memory is usually evaluated using the EAM test. In this study, either spatial memory or working memory performance was greatly improved by *Sal* treatment in rats administered with long-term EtOH. Additionally, according to the most popular theory in recent times, memory improvement by *Sal* treatment is likely linked to alteration in gut-brain function.

Gut microbiota are an integral part of the human body and affect human health and disease (Cockburn and Koropatkin, 2016). Studies have shown that alcohol consumption can lead to changes in the composition of gut microbes and affect immune factors, immune system tolerance (Wang et al., 2022; Ye et al., 2022), and immune system function, which can lead to learning and memory impairment (Bishnoi et al., 2022). *Sal* plays a beneficial immunomodulatory role by regulating immune cell differentiation, inflammatory signaling pathway activation, and inflammatory factor secretion to reduce inflammatory damage in various diseases (Drayton et al., 2006; Wang et al., 2013). For example, Saldiogenin, a major component of *Rhodiola rosea L*, can reduce excessive inflammatory responses caused by asthma or cerebral ischemia by regulating the balance of helper T cells (Th1/Th2) [8] or macrophage polarization (Liu et al., 2018; Wang et al., 2018). These studies reflect the modulatory effects of Saldiogenin on various immune cells.

At this stage, the use of rats to model diseases of alcohol has been increasingly refined, and the various diseases caused by alcohol have been increasingly clearly studied (García-García et al., 2021; Crofton et al., 2022; Maccioni et al., 2022). However, changes in alcohol-induced intestinal flora and studies on brain-related brain regions are relatively rare. In the present study, the intestinal contents of rats were collected by continuous administration of *Sal* intervention. The flora of rat intestinal contents could be analyzed more accurately. By macrogenome sequencing analysis, we found significant differences in the abundance and diversity of the flora among the Model, *Sal*, and CON groups. This finding is consistent with the findings of Wang et al. (2023). The PCoA results showed that the composition of the flora was similar between the *Sal* and Model groups, but the relative abundance was differed. At the phylum level, the Model group had a higher relative abundance of the thick-walled phylum and a lower relative abundance of the *Actinobacteria phylum*, whereas the relative abundance of *Bifidobacterium* was lower. *Sal* intervention significantly increased the relative abundance of *Bifidobacterium*.

*Bifidobacterium* can pre-prevent alcoholic liver disease by modulating the intestinal microbiota in mice with chronic alcohol intake (Aldridge et al., 2022). To find the differential microorganisms between the *Sal* and Model groups using linear discriminant analysis, this study found that the significantly different families in the Model and *Sal* groups were *E. coli* and *Bifidobacterium* families, respectively. Numerous studies have found that *E. coli* can cause diarrhea and other symptoms in various organisms, including humans and mice (Shahbazi et al., 2021; Yu et al., 2023). Similarly, in this study, diarrheal symptoms were observed in the Model group. Additionally, *Bifidobacterium*, as one of the important probiotics, can regulate the balance of intestinal flora, inhibit tumor growth, and regulate immune function in the intestines (Li et al., 2022; Ren et al., 2022). In this study, we found that *Sal* significantly increased the relative abundance of *Bifidobacteria* and decreased the relative abundance of *E. coli* in rat intestine, thus acting as a regulator of the intestinal flora. This finding is consistent with the results of a previous study (Miyazaki et al., 2010). Therefore, *Sal* may play an important role in regulating intestinal flora imbalance. This finding is consistent with previous findings on the effects of other herbal medicines on intestinal flora (Mei et al., 2022; Xu et al., 2022; Chen et al., 2023). Functional enrichment analysis of the macrogenome showed that *Sal* affects the endocrine system and environmental adaptations by regulating intestinal flora.

A growing number of studies have shown that alterations in gut microbiota are closely associated with the development of neurological diseases (Hang et al., 2022), and alcohol consumption can lead to pathophysiological changes in several brain regions (Hansen et al., 2020; Varodayan et al., 2022). To further investigate whether *Sal* regulates the gut–brain axis through gut microbiota, we performed transcriptional sequencing analysis of hippocampal tissues. Transcriptional sequencing revealed 26 differentially expressed genes in the *Sal* group compared to the model group. They included 15 genes with elevated expression (Olr1694, Ca3, Synpo2, etc.) and 11 genes with decreased expression (Ch25h, Tubb2b, Tomm7, etc.). One study found that by rapidly increasing carbonic anhydrase (CA) concentrations in the cortex and hippocampus (two brain regions involved in memory processing), the extracellular signal-regulated kinase (ERK) pathway in the cortex and hippocampus—a key step in memory formation—could be rapidly increased (Canto de Souza et al., 2017).

Growing evidence indicate that brain carbonic anhydrase (CA) is a key regulator of cognition, especially in recognition and aversive memory. A study has found that injecting inhibitors of carbonic anhydrase in the hippocampal CA1 region or mPFC impairs short-term social recognition memory (Schmidt et al., 2022). The aforementioned study is consistent with the present study, in which the Ca3 gene expression in the hippocampus was increased by *Sal* intervention, which in turn had the effect of improving memory. Abnormalities in β-microtubulin (Tubb2b) have been suggested to be probably associated with the pathophysiology of schizophrenia and have a unique role in neuronal differentiation and cell viability. In patients with schizophrenia, Tubb2b protein expression is reduced in the anterior cingulate cortex and increased in the dorsolateral prefrontal cortex; however, it remains unchanged in the superior temporal gyrus or hippocampus. In contrast, alcohol consumption can lead to cytoskeletal changes (Moehle et al., 2012). In the present study, we found that *Sal* intervention reduced hippocampal Tubb2b gene expression, which provides an idea to study memory impairment from a genetic perspective.

A GSEA of gene function based on the GO database for the *Sal* and Model groups revealed 652 significant GO terms between the two groups. The top five terms included mitochondrial inner membrane, translation, immune response, structural constituents of ribosome, and mRNA splicing, *via* spliceosome. The immune system is an important system that performs immune response and immune function, and alcohol abuse can lead to impairment of the immune system, which can be inherited by offspring through the maternal generation, resulting in severe immune deficiency (Bake et al., 2021). In the present study, we found that *Sal* is likely to improve alcohol-induced memory impairment by regulating hippocampal immune response, which provides a scientific basis for investigating the association between immune response and alcohol exquisite memory impairment.

Notably, we observed that the upregulation of Tomm7 and Klhl40 mRNA in rat hippocampus exhibited a positive regulatory role on behavioristics. Tomm7 is a gene that encodes a subunit of mitochondrial outer membrane translocase and is involved in the transport and stability of mitochondrial proteins, and klhl40 is a gene that encodes a protein containing Kelch repeat domains and is involved in muscle development and function. Although none direct evidence, there still several hints that changes in the expression of tomm7 and klhl40 genes in the hippocampus of the alcohol-dependent rat model are associated with gut microbiota. One study found that acute ethanol treatment can induce neurodegeneration in cultured hippocampal neurons, leading to mitochondrial dysfunction, calcium processing defects, and synaptic damage in hippocampal neurons. This means that mitochondrial-related genes (such as tomm7) also play a role in this process (Pérez et al., 2020). Another study found that during the withdrawal period after alcohol exposure, the expression of miRNAs (microRNAs) and mRNAs (messenger RNAs) in the rat hippocampus area changed significantly, including some genes related to muscle development. This may mean that muscle-related genes (such as klhl40) also play a role in this process (Engen et al., 2015). Of course, these are only speculations and are not enough to prove a direct or indirect association between tomm7, klhl40 genes and gut microbiota. More experimental data and analysis are needed to determine. Therefore, we further verified the variation of tomm7 and klhl40 in hippocampus by real-time PCR to ensure the viability of these hints.

Further, the administration of Sal leading to a downregulation of tomm7, as well as the positive correlation with the abundance of *Alistipesindistinctus*, *Lactobacustaiwanensis*, *Lactobacusparagi*, and *Lactobacusparagillusjohnsonii* in the intestine of the *Sal* group. Some studies suggest that *Alistipes indistinctus* can interact with other gut microbiota to influence gut barrier, immune system, and metabolism (Wang et al., 2021). None clear research on the physiological functions of *Lactobacustaiwanensis*. However, *Lactobacillus crispatus* is a common probiotic that mainly inhabits the vagina and intestine. It can produce hydrogen peroxide and has antibacterial, anti-inflammatory, and immune-regulatory effects (Lopez-Vicchi et al., 2020). *Lactobacusparagi* and *Lactobacusparagillusjohnsonii* are the type of lactic acid bacteria, belonging to the *Lactobacillus genus*. Their physiological functions have not been clearly studied. However, lactic acid bacteria generally have functions such as fermentation, lowering pH, inhibiting harmful bacteria, producing vitamins, and promoting digestion (Zhu et al., 2019). In our study, we first initiated the physiological functions of *Lactobacustaiwanensis*, *Lactobacusparagillusjohnsonii* and *Lactobacusparagi*, the type of *Lactobacillus* during tomm7-related Sal treatment in alcoholic memory impairment. There is a certain association between gut *Lactobacillus* and alcohol-induced memory impairment. Some studies suggest that supplementing with *Lactobacillus* can improve the composition and function of gut microbiota, thereby alleviating alcohol-induced damage to hippocampal neurons and cognitive function (Bloemendaal et al., 2021; Sun et al., 2022). Although none direct evidence reporting the association between tomm7 and *Lactobacillus*. However, some studies identified that *Lactobacillus* could regulate the functions of the immune and nervous systems by producing outer membrane vesicles and soluble factors. These factors may affect the transport and stability of mitochondrial proteins, indirectly affecting the function of tomm7 (Sun et al., 2015; Di Cerbo et al., 2016; Mata Forsberg et al., 2019). Our research suggests that Sal can affect the expression of tomm7 in the hippocampus, and therefore tomm7 can be used as a therapeutic target to improve alcohol-induced memory impairment, with Sal being a potential drug under this pathological condition. Additionally, the *Alistipesindistinctus*, and three *Lactobacillus* of *Lactobacustaiwanensis*, *Lactobacusparagi*, and *Lactobacusparagillusjohnsonii Lactobacillus* represented can be used as dominant probiotics to affect hippocampal function and may serve as potential target microbiota for disease regulation and functional improvement, providing a compelling evidence for the translation of future drugs and the application of microbiota.

## Conclusion

Sustained intervention with *Sal* improves alcoholic memory dysfunction. Furthermore, this study revealed that *Sal* can alter the diversity and composition of gut microbiota and affect the expression of a large number of genes in the hippocampus of rats with alcohol-induced memory impairment. To our knowledge, this study is the first to report the effects of *Sal* intervention on gut microbiota and hippocampal transcriptome in rats with alcohol memory impairment. This study provides an important scientific basis for the role of the gut–brain axis in inducing alcohol memory impairment and elucidates the therapeutic role of *Sal* in the treatment of alcoholic memory impairment.

## Data availability statement

The datasets presented in this study can be found in online repositories. The name of the repository and accession number can be found at: China National Microbiology Data Center (NMDC); NMDC0000192.

## Ethics statement

The animal study was reviewed and approved by the Ethics Committee of Qiqihar Medical University.

## Author contributions

YJ, RZ, PL, and XL designed the experiments. YJ, ZZ, TW, LL, TL, XL, and DX performed the experiments. YJ, SW, PL, and XL performed the statistical analysis. RZ, PL, DX, ZZ, and YJ wrote the manuscript. All authors contributed to this article and approved the submitted version.

## Funding

This study was funded by the National Natural Science Foundation of China (82104173), and Qiqihar Academy of Medical Sciences, Grant No. QMSI2019M-09; Heilongjiang Provincial College Students Innovation and Entrepreneurship Project, Grant No. 201911230056; The Science Research Foundation of Qiqihar Medical Institute No. 2021-ZDPY-014; The Science Research Foundation of Qiqihar city, China No. LHYD-2021015; The Science Research Foundation of Qiqihar city, Qiqihar city, China No. LHYD-202011.

## Conflict of interest

The authors declare that the research was conducted in the absence of any commercial or financial relationships that could be construed as a potential conflict of interest.

## Publisher’s note

All claims expressed in this article are solely those of the authors and do not necessarily represent those of their affiliated organizations, or those of the publisher, the editors and the reviewers. Any product that may be evaluated in this article, or claim that may be made by its manufacturer, is not guaranteed or endorsed by the publisher.

## Footnotes

^1^ https://www.bioincloud.tech
