# Supplementary material for: Salidroside ameliorates memory impairment following long-term ethanol intake in rats by modulating the altered intestinal microbiota content and hippocampal gene expression
Source: Front Microbiol. 2023 Jun 9;14:1172936. doi: 10.3389/fmicb.2023.1172936 (PMC10288325; doi:10.3389/fmicb.2023.1172936)
Supplement: Supplementary file 1 [file Data_Sheet_1.pdf]

Salidroside ameliorates memory impairment following long-term ethanol intake in rats by modulating the altered intestinal microbiota content and hippocampal gene expression

Yu Jiao [1†], **Zhenglin Zhao** [2†], Xin Li [3], Lulu Li [2], Dan Xiao [4,5], Siyuan Wan [6], Tong Wu [1], Tong Li [1], **Ping Li** [1*], **Rongjie Zhao** [1*]

[1] Department of Psychiatry, Qiqihar Medical University, Qiqihar, Heilongjiang, China,

[2] Department of Biochemistry, Qiqihar Medical University, Qiqihar, Heilongjiang, China,

[3] Department of Psychiatry, The Fourth Affiliated Hospital of Qiqihar Medical University, Qiqihar, Heilongjiang, China,

[4] School of Medicine and Health, Harbin Institute of Technology, Harbin, Heilongjiang, China,

[5] Department of Medicine and Health, Zhengzhou Research Institute of Harbin Institute of Technology, Zheng Zhou, He Nan, China,

[6] Department of Preventive Medicine, Qiqihar Medical University, Qiqihar, Heilongjiang, China.

*** Correspondence:**

Ping Li, lipingchxyy@163.com;

Rongjie Zhao, zhao_rongjie@yahoo.com.

Yu Jiao [1†], Zhenglin Zhao [2†]These authors contributed equally to this work and share first authorship

Supplementary tables and Figures

Table S1. Macrogenome sequencing quality control results of CON group, Sal group and Model group(n=6).

Sample ID	InsertSize(bp)	RawReads(#)	%GC	Raw Q20(%)	Raw Q30(%)	Clean Reads(#)	Cleaned(%)	Clean Q20(%)	Clean Q30(%)
C01	350	29409307	45	97.54	93.15	28424192	96.65	98.49	94.61
C02	350	30311132	46	97.49	93.13	25148501	82.97	98.5	94.68
C03	350	33847979	50	97.39	92.94	32327192	95.51	98.43	94.54
C04	350	29923538	49	97.41	92.97	28721978	95.98	98.44	94.55
C05	350	27808963	47	97.57	93.26	25014066	89.95	98.5	94.68
C06	350	31967297	49	97.39	92.95	30098964	94.16	98.44	94.56
M01	350	19956845	33	97.62	92.84	19121663	95.82	98.43	94.11
M02	350	29144252	45	97.51	93.14	27782063	95.33	98.5	94.65
M03	350	26821486	48	97.49	93.09	25831041	96.31	98.46	94.58
M04	350	27950494	46	97.63	93.35	26919710	96.31	98.55	94.78
M05	350	27119279	47	97.52	93.15	25645751	94.57	98.49	94.64
M06	350	31243095	45	97.59	93.26	29868807	95.6	98.52	94.71
S01	350	27433392	45	97.46	92.97	25310594	92.26	98.45	94.48
S02	350	19910940	47	97.32	92.79	17087379	85.82	98.4	94.46
S03	350	20196123	48	97.28	92.68	19375054	95.93	98.37	94.39
S04	350	21782982	48	97.34	92.82	19967168	91.66	98.39	94.42
S05	350	21733699	46	97.4	92.9	20835671	95.87	98.43	94.49
S06	350	21294689	49	97.42	92.97	19690160	92.47	98.45	94.58

Table S2. Transcriptome sequencing quality control results of CON group, Sal group and Model group(n=6).

sample id	total reads	total bases	q20 bases	q30 bases	q20 rate	q30 rate	gc content
C01	44826036	6458050391	6294863717	5979463418	0.974731	0.925893	0.477972
C02	47567584	6857417764	6692118408	6367327724	0.975895	0.928531	0.48291
C03	46300010	6666575262	6506629561	6196726040	0.976008	0.929522	0.49242
C04	44449660	6411101942	6254753725	5946207459	0.975613	0.927486	0.469155
C05	45337554	6537692946	6379727464	6069638291	0.975838	0.928407	0.482851
C06	44838124	6454868279	6311679863	6023275656	0.977817	0.933137	0.471465
M01	42006190	6058155238	5909542055	5621388917	0.975469	0.927904	0.484789
M02	43885278	6317874599	6170874069	5882813137	0.976733	0.931138	0.495705
M03	45110372	6480360708	6311493896	5991833889	0.973942	0.924614	0.497646
M04	54579352	7846741383	7649707149	7271521998	0.97489	0.926693	0.494489
M05	43431690	6241472434	6035806726	5674120309	0.967049	0.9091	0.495197
M06	45569406	6543675968	6378096835	6059647477	0.974696	0.926031	0.493884
S01	47493268	6852209167	6690022189	6373520694	0.976331	0.930141	0.498258
S02	44610658	6436286739	6284866726	5989125194	0.976474	0.930525	0.496002
S03	52630166	7580741470	7403680198	7053589506	0.976643	0.930462	0.486185
S04	48083928	6916637077	6756199385	6439480443	0.976804	0.931013	0.478821
S05	46241984	6659894443	6471580602	6115487010	0.971724	0.918256	0.489782
S06	45772280	6602564502	6424067937	6086597543	0.972966	0.921854	0.482432

Figure S1

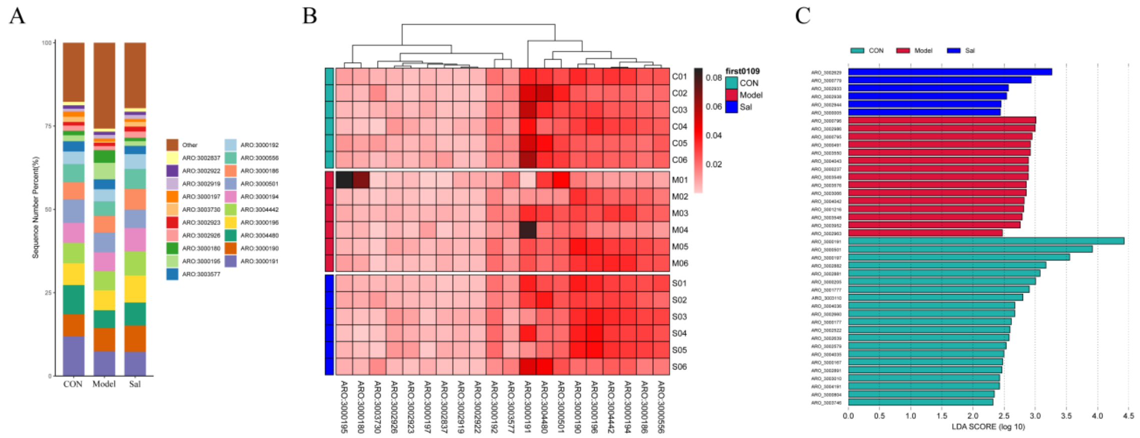

Figure S1 Histogram, clustering heat map and LEfSe analysis of intestinal resistance genes among CON group, Sal group and Model group.

Figure S2

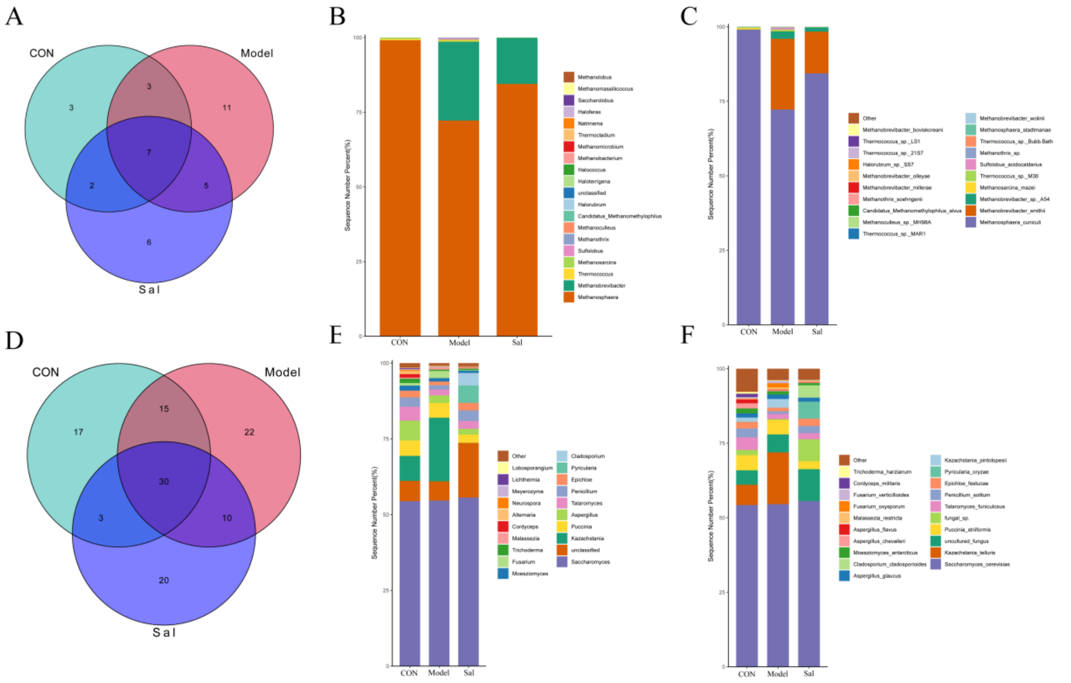

Figure S2.Diversity of intestinal archaea and fungi (S2-A, S2-D) and relative abundance histograms at the genus level and species level (S2-BC,S2-EF) among the CON group, Sal group and Model group.

Figure S3

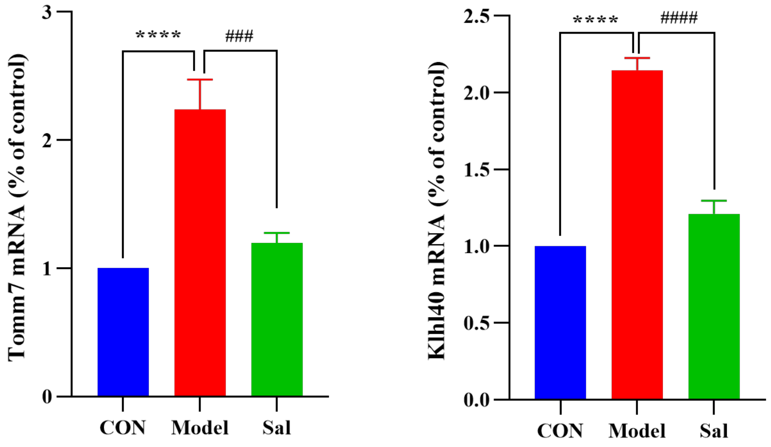

Figure S3.Relative mRNA expression of *Tomm7* and *Klhl40* in hippocampus (**** $P < 0.0001$ (CON vs Model); $^{\#\#\#}$ $P < 0.001$ (Model vs Sal); $^{\#\#\#\#}$ $P < 0.0001$ (Model vs Sal)).
